# Combining the Risk: The Poly-Environmental Risk Score and Psychotic Symptoms in Adolescents

**DOI:** 10.1093/schbul/sbaf046

**Published:** 2025-04-14

**Authors:** Diandra C Bouter, Susan J Ravensbergen, Nita G M de Neve-Enthoven, Sibel Ercan, Benno Bakker, Mark H de Jong, Witte J G Hoogendijk, Nina H Grootendorst-van Mil

**Affiliations:** Department of Psychiatry, Erasmus MC, University Medical Center Rotterdam, 3000 CA, Rotterdam, The Netherlands; Department of Psychiatry, Erasmus MC, University Medical Center Rotterdam, 3000 CA, Rotterdam, The Netherlands; Department of Psychiatry, Erasmus MC, University Medical Center Rotterdam, 3000 CA, Rotterdam, The Netherlands; Department of Psychiatry, Erasmus MC, University Medical Center Rotterdam, 3000 CA, Rotterdam, The Netherlands; Epidemiological and Social Psychiatric Research Institute (ESPRi), Department of Psychiatry, Erasmus MC, University Medical Center Rotterdam, 3000 CA, Rotterdam, The Netherlands; Parnassia Psychiatric Institute, 3009 AM, Rotterdam, The Netherlands; Epidemiological and Social Psychiatric Research Institute (ESPRi), Department of Psychiatry, Erasmus MC, University Medical Center Rotterdam, 3000 CA, Rotterdam, The Netherlands; Yulius Mental Health, 3300 BA, Dordrecht, The Netherlands; Department of Psychiatry, Erasmus MC, University Medical Center Rotterdam, 3000 CA, Rotterdam, The Netherlands; Department of Psychiatry, Erasmus MC, University Medical Center Rotterdam, 3000 CA, Rotterdam, The Netherlands; Epidemiological and Social Psychiatric Research Institute (ESPRi), Department of Psychiatry, Erasmus MC, University Medical Center Rotterdam, 3000 CA, Rotterdam, The Netherlands

**Keywords:** epidemiology, psychopathology, cohort, at risk

## Abstract

**Background and Hypothesis:**

Psychotic symptoms are common in adolescents and predictive of psychiatric disorders. Numerous risk factors have been shown to precede psychiatric disorders. However, investigating individual risk factors does not account for the cumulative effect these risk factors may have. Therefore, we combined well-researched environmental risk factors for psychotic disorder in a composite measure: the poly-environmental risk score (PERS).

**Study Design:**

Risk factors were assessed in a cohort of 801 adolescents (aged 15) at risk for psychopathology. Binarized risk factors included winter birth, low gestational age, low birth weight, ethnic minority status, urban living environment, cannabis use, victim of bullying, emotional abuse, physical abuse, sexual abuse, high paternal age, parental severe mental illness, parental divorce, and parental death. The PERS was weighted with the log odds derived from recent meta-analyses. At age 18, self-reported psychotic experiences (PE) and clinician-rated psychotic symptoms (PS) were assessed. This updated PERS was compared to previous PERS models, which included fewer risk factors and different weightings.

**Study Results:**

The PERS was associated with PE and PS. Specifically, a PERS between 3 and 4, and PERS > 4 corresponded with a 2.2- and 5.2-fold increase in the odds of psychotic symptoms in late adolescence. The updated 14-item PERS performed better compared to previous compositions of the PERS.

**Conclusions:**

A composite score of childhood and adolescent risk factors measured at age 15 was associated with psychotic symptoms at age 18. Future research should consider the cumulative effect of risk factors when examining the determinants of psychopathology.

## Introduction

The onset of psychiatric disorders is generally preceded by exposure to various risk factors. Umbrella reviews indicate a multitude of associations between individual risk factors and various psychiatric disorders, including neurodevelopmental, mood, anxiety, and psychotic disorders.^1–8^ Risk factors frequently co-occur and most are associated with multiple disorders.^9–11^

To better represent the complexity of psychopathology and improve prediction accuracy, researchers have therefore started to combine risk factors into an aggregated risk score.^12,13^ This aggregated exposome score, known as the poly-environmental risk score (PERS), is modeled after the polygenic risk score (PRS), which calculates genetic liability for certain disorders.^14^ The PERS integrates various environmental risk factors, weighted by meta-analytic odds ratios, to compute a single estimate of the combined risk.

Studies have demonstrated that the PERS can identify environmental liability for conversion to psychotic disorders in a high-risk population,^12^ assess the risk of psychosis,^13,15,16^ and distinguish between cases and controls.^17–19^ Combining risk factors is not only important because of the potential additive effects of multiple risk factors but also because of the clinical implications. Previous studies have shown that PERS is related to the age of onset of psychotic disorders^20^ and the general functioning of patients.^21^ However, few studies have examined whether the PERS is associated with problems prior to the onset of psychotic disorders. One study found that the PERS was associated with psychotic experiences (PE) in adults,^22^ but no association was found in a study with adolescents.^23^ In children, the PERS was associated with schizotypy profiles.^24^ Studies used different compositions of the PERS, with different weightings, and often not including specific types of adverse life events or parental factors such as severe mental illness.

In this study, we propose using the PERS to investigate its association with psychotic experiences and psychotic symptoms in a population-based high-risk cohort of adolescents. Participants were assessed for lifetime risk factors at age 15 and for psychotic experiences and symptoms at age 18. We examined whether the PERS was indicative of self-reported psychotic experiences and clinician-rated psychotic symptoms in late adolescence. Specifically, we used an updated PERS that included 14 risk factors weighted with the most recent meta-analytical odds ratios, and compared this to previously reported PERS models.

## Methods

Adolescents were assessed as part of the iBerry (Investigating Behavioral and Emotional Risk in Rotterdam Youth) Study. This population-based cohort study follows 1.022 adolescents into young adulthood to investigate how subclinical symptoms develop into psychiatric disorders. Adolescents were selected based on their self-reported emotional and behavioral problems (Strengths and Difficulties Questionnaire-Youth, SDQ-Y^25^) at age 13. Adolescents with a high score (top 15%) were oversampled over adolescents with a lower score (lowest 85%) with a 2.5:1 ratio.^26^ This resulted in a cohort at risk of developing psychopathology. At the mean age of 15, the adolescents participated in the extensive baseline measurement,^26^ at the mean age of 18 adolescents participated in the first follow-up measurement (T1).^27^ The study is conducted in the greater Rotterdam area, including adolescents from different urbanized areas with varying socioeconomic backgrounds. The study was approved by the Medical Ethical Research Committee of the Erasmus Medical Center and all participants signed informed consent before participating.

## Measurements

### Psychotic Experiences and Symptoms

Psychotic experiences were assessed with the 16-item Prodromal Questionnaire.^28^ The questionnaire assesses hallucinatory and delusional experiences as well as negative symptoms. A cut-off score of 6 was used to identify adolescents who report a significant number of PE, indicating a heightened risk for developing psychotic disorders.^28^

Psychotic symptoms were assessed with a semistructured interview conducted by a trained researcher, the Mini-International Neuropsychiatric Interview for Children and Adolescents (MINI-KID^29^). The MINI-KID assesses 5 delusional symptoms as well as visual and auditory hallucinations. PE and PS were both assessed at T1. A combined measure was created by selecting all adolescents who scored above the cut-off of 6 for PE and/or reported PS, to create a PE/PS group.

### Environmental Risk Factors

An overview of the assessed risk factors with their corresponding odds ratios is presented in Table 1. The odds ratios were selected from the most recent meta-analyses available. All known risk factors that were measured in the cohort were included. For some measured variables, we did not identify a meta-analysis supporting the variable as a risk factor. These factors (socioeconomic status, global functioning, psychopathology, suicidality, substance use other than cannabis use, physical health, and puberty timing) were therefore not included in the PERS. All 14 included risk factors were assessed during the baseline measurement, when adolescents were on average 15 years old. The season of birth was determined on the date of birth of the adolescent and based on categorized according to the astronomical seasons, starting on the 21st of the month. Urbanicity was determined based on the house address (including home number) of the adolescent. Ethnic background was determined by the country of birth of the parents. Adolescents were categorized in the ethnic minority category if one or both of the parents were born in a non-Western country defined as any country outside Europe, North America, Australia, or New Zealand. Adolescents filled out questionnaires to indicate the lifetime use of cannabis and whether they had experienced bullying (4 items). Emotional abuse was measured with the Parent–Child Conflict Tactics Scale (CTSPC^38^) completed by the adolescents about both parents and by one of the parents about the adolescent. If at least one of the informants scored “often” on one or more items of the psychological aggression scale emotional abuse was counted as a risk factor. Parental death, parental divorce, physical abuse, and sexual abuse were assessed with the childhood adversity interview from the TRAILS Study (Tracking Adolescents Individual Lives Survey^39^). The parent indicated whether the adolescents had experienced these adverse life events. The Mini-International Neuropsychiatric Interview—Plus (MINI-Plus^40^) was conducted to assess the lifetime DSM-IV diagnoses of one of the parents. Severe mental illness was defined as having met the criteria for major depressive disorder, bipolar disorder, and/or psychotic disorder. In questionnaires, parents reported on the adolescent’s gestational age, adolescent’s birth weight, and paternal age at birth. All risk factors were binarized as present or absent for analyses (see Table 1 for criteria).

**Table 1.** Environmental Risk Factors with Their Meta-analytic Odds Ratios

Risk factor	OR	Log OR	Source	Cut-off used to determine presence of risk factor
Winter birth	1.05	0.049	Davies et al., 2020^30^	Born between December 21st and March 20th
Low gestational age	1.35	0.300	Davies et al., 2020^30^	Gestational age < 37 weeks
Low birth weight	1.53	0.425	Davies et al., 2020^30^	Birth weight < 2500 grams
Ethnic minority status	1.82	0.599	Henssler et al., 2020^31^	Non-western country of birth of at least one parent
Urban living area	2.39	0.871	Vassos et al., 2012^32^	Surrounding home address density of > 1000 addresses per square kilometer
Used cannabis	1.97	0.678	Marconi et al., 2016^33^	Has used cannabis
Has been bullied	2.28	0.824	Pastore et al., 2022^34^	Has been bullied multiple times
Emotional abuse	3.40	1.224	Varese et al., 2012^35^	Reported as “often” by child or parent
Physical abuse	2.95	1.082	Varese et al., 2012^35^	Present
Sexual abuse	2.38	0.867	Varese et al., 2012^35^	Present
High paternal age	1.28	0.247	Davies et al., 2020^30^	Paternal age > 35 years at time of birth
Parental divorce	1.53	0.425	Ayerbe et al., 2020^36^	Present
Parental severe mental illness	3.94	1.371	Rasic et al., 2014^37^	Lifetime DSM diagnosis of major depressive, bipolar, or psychotic disorder of at least one parent
Parental death	1.24	0.215	Pastore et al., 2022^34^	Present

### Missing Values

Missing values on psychotic experiences and symptoms were not imputed. The environmental risk factors for bullying, abuse, parental severe mental illness, parental divorce, and parental death were missing for 7%-12% of the adolescents. Birth weight, gestational age, and paternal age were missing for approximately 30% of the adolescents. All other risk factors had minimal missing data (0%-2%). Missing data on risk factors were imputed in IBM SPSS Statistics using multiple imputation with an iterative Markov Chain Monte Carlo method (MCMC). Data were assumed to be missing at random, 15 imputed datasets were created using the fully conditional specification (FCS) method.^41^ The imputed analyses were pooled using Rubin’s rules and compared to complete-case analyses.

### Data Analysis

First, descriptive statistics were obtained and correlations between risk factors were assessed. Logistic regression analyses were used to examine the associations between the individual risk factors and PE and PS. A simple environmental risk score (ERS) was calculated by summing the 14 binarized risk factors (possible range 0-14). The weighted PERS was calculated by adding the log odds ratios for the present risk factors for each adolescent (maximum of 9.18). The PERS was then multiplied by 14 to facilitate the interpretation of the coefficients. Logistic regression analyses were used to examine if the ERS and PERS were associated with PE and PS. Next, adolescents were categorized into groups based on the absolute PERS score (<1, <2, <3, <4, and ≥4) to estimate the same models and examine potential dose–response effects. All analyses were adjusted for the age and sex of the adolescent and first performed for the total PE/PS group (yes on either or both PE and PS) and then specifically for PE (self-report) and PS (clinician-rated). We used the Benjamini–Hochberg method to correct for multiple testing.

#### Sensitivity Analyses.

Two alternative PERSs from previous studies were calculated to compare with the performance of the updated 14-item PERS in predicting PE and PS. One 9-factor PERS in line with the study of Padmanabhan from 2017 (P-PERS,^12^) was calculated by summing the log odds for winter or spring birth (0.068), birth weight ≤ 2.5 kg (0.69), urban living (0.54), cannabis use (0.56), physical abuse (1.08), sexual abuse (0.87), paternal age over 35 years (0.25), and parental death (0.53). We were unable to include neglect as the ninth risk factor as data on the subject was not available.

Next, the 6-factor Maudsley ERS from 2020 (M-PERS,^13^) was calculated by summing 6 risk factors: ethnic minority status (+2.5) or non-minority status (as native, −0.5), urbanicity low (−1.5) or high (+1), paternal age 40-50 years (+0.5), birth weight ≤ 2.5 kg (+2), cannabis exposure (no exposure, −1; exposure +3), and any childhood adversity (+2.5).

Furthermore, we examined whether the associations would remain if we excluded familial risk from the PERS as severe mental illness of the parent has both a genetic and an environmental effect.

Lastly, linear regression analyses to examine the associations between the composite score and the total score of PE were also included as a sensitivity analysis. All analyses were performed using IBM SPSS Statistics, version 28.^42^

## Results

The study sample consisted of 801 adolescents who provided data on either or both PE and PS at follow-up, as described in Table 2. Adolescents had a mean age of 18.1 years, 53.6% were female, and most reported a (pre)vocational education level (low, 52.8%). The most prevalent risk factors were living in an urban area, paternal age at birth over 35 years, and having a parent with severe mental illness. PE were reported by 115 adolescents (14.5%) and PS by 110 adolescents (15.1%). Overall, 172 adolescents (21.5%) reported either PE, PS, or both. PE and PS data were missing for 1.2% and 9.0% of the adolescents, respectively.

**Table 2.** Sociodemographic Characteristics and Prevalence of Environmental Risk Factors for the Total Cohort and Stratified for Adolescents with and without Psychotic Experiences and Psychotic Symptoms at Age 18

	Total cohort (*n* = 801)	PE/PS group (*n* = 172)	No PE/PS group (*n* = 629)
Age at baseline, years (*M*, *SD*)	14.94 (0.91)	14.90 (0.96)	14.94 (0.89)
Age at follow-up, years (*M*, *SD*)	18.07 (0.85)	17.99 (0.83)	18.09 (0.86)
Sex female (*n*, %)	429 (53.6)	92 (53.5)	337 (53.6)
Educational level at age 18 (*n*, %)			
Low	414 (52.8)	104 (62.3)	310 (50.2)
Medium	211 (26.9)	39 (23.4)	172 (27.9)
High	159 (20.3)	24 (14.4)	135 (21.9)
Low socioeconomic status (*n*, %)	116 (16.0)	29 (19.3)	87 (15.1)
			
**Risk factors (*n*, %)**			
Winter birth	166 (20.7)	33 (19.2)	133 (21.1)
Gestational age < 37 weeks	49 (8.6)	7 (6.4)	42 (9.1)
Birth weight < 2500 gram	40 (7.4)	9 (8.5)	31 (7.2)
Ethnic minority status	140 (17.5)	42 (24.6)	98 (15.6)
Urban living area	630 (78.7)	138 (80.2)	492 (78.2)
Used cannabis	53 (6.6)	11 (6.4)	42 (6.7)
Has been bullied	82 (11.2)	32 (20.5)	50 (8.7)
Emotional abuse	167 (22.3)	48 (29.6)	119 (20.3)
Physical abuse	162 (22.9)	42 (27.8)	120 (21.6)
Sexual abuse	40 (5.6)	10 (6.6)	30 (5.3)
Paternal age > 35 years	209 (40.4)	37 (37)	172 (41.2)
Parental divorce	177 (24.2)	50 (31.8)	127 (22.2)
Parental severe mental illness	220 (30.4)	63 (40.1)	157 (27.7)
Parental death	6 (0.8)	3 (1.9)	3 (0.5)

Educational level was missing for 17 adolescents. Number of missings per risk factor: gestational age (229), birth weight (262), ethnic background (3), SES (76), cannabis use (3), bullying (72), emotional abuse (53), physical abuse (95), sexual abuse (85), paternal age (284), parental divorce (71), parental severe mental illness (78), parental death (70).

Supplementary Table S1 shows the correlations between the risk factors. The majority of risk factors were uncorrelated. With the exception of the correlation between gestational age and birth weight (*r* = .57, *P* ≤ .001), significant correlations were very low (coefficients ranging from *r* = .04 to .17).

Individual linear regression analyses showed that not all individual risk factors were associated with PE and/or PS (Table 3). Ethnic minority status, parental divorce, and parental severe mental illness were associated with PE at age 18. Ethnic minority status, bullying, emotional abuse, and parental divorce were associated with PS at age 18.

**Table 3.** ORs from Logistic Regression Analyses for Each Individual Environmental Risk Factor, a Combined Simple Sum Score and the PERS in Association with Psychotic Experiences and Psychotic Symptoms in Adolescents

	Total PE/PS group		Psychotic experiences (*n* = 791)		Psychotic symptoms (*n* = 679)	
	(*n* = 801)					
	OR (95% CI)	*P*	OR (95% CI)	*P*	OR (95% CI)	*P*
**Environmental risk factors**						
*Adolescent factors*						
Winter birth	0.91 (0.59;1.40)	.6655	0.86 (0.51;1.43)	.5508	1.00 (0.61;1.65)	.9925
Gestational age	0.86 (0.43;1.72)	.6708	0.99 (0.43;2.25)	.9765	0.93 (0.42.;2.06)	.8483
Birth weight	1.09 (0.57;2.08)	.7983	1.12 (0.52;2.43)	.7721	1.14 (0.53;2.45)	.7373
Ethnic minority status	**1.88 (1.24;2.85)**	**.0031**	**1.82 (1.13;2.95)**	**.0147**	**1.92 (1.19;3.12)**	**.0080**
Urban living area	1.11 (0.73;1.70)	.6151	1.16 (0.71;1.92)	.5542	1.23 (0.73;2.07)	.4471
Used cannabis	0.97 (0.49;1.94)	.9410	0.62 (0.24;1.60)	.3239	1.08 (0.49;2.38)	.8500
Has been bullied	**2.42 (1.52;3.86)**	**.0002**	1.56 (0.89;2.76)	.1225	**2.56 (1.50;4.36)**	**.0006**
Emotional abuse	**1.63 (1.10;2.42)**	**.0148**	1.51 (0.95;2.39)	.0797	**1.85 (1.18;2.90)**	**.0076**
Physical abuse	1.50 (1.00;2.25)	.0504	1.46 (0.92;2.33)	.1068	1.81 (0.93;3.51)	.0786
Sexual abuse	1.39 (0.76;2.54)	.2788	1.20 (0.58;2.50)	.6205		
*Parental factors*						
Paternal age	0.83 (0.55;1.26)	.3782	0.81 (0.49;1.34)	.4114	0.70 (0.41;1.19)	.1834
Parental divorce	**1.62 (1.09;2.41)**	**.0183**	**1.67 (1.06;2.62)**	.0269*	**1.59 (1.01;2.52)**	.0466*
Parental severe mental illness	**1.63 (1.13;2.35)**	**.0097**	**1.78 (1.17;2.71)**	**.0067**	1.29 (0.83;2.01)	.2513
Parental death	2.45 (0.55;10.96)	.2417	3.97 (0.88;17.98)	.0740	2.51 (0.45;13.96)	.2930
						
**PERS score**						
ERS (simple sum)	**1.23 (1.11;1.36)**	**.0001**	**1.20 (1.06;1.35)**	**.0029**	**1.24 (1.10;1.41)**	**.0006**
						
PERS (continuous)	**1.38 (1.20;1.60)**	**<.0001**	**1.32 (1.12;1.55)**	**.0011**	**1.45 (1.22;1.72)**	**<.0001**
						
PERS, score between 1 and 2	1.41 (0.82;2.45)	.2182	**2.03 (1.05;3.94)**	.0358*	1.15 (0.58;2.28)	.6946
PERS, score between 2 and 3	1.42 (0.79;2.56)	.2471	1.77 (0.85;3.69)	.1264	1.45 (0.71;2.96)	.3025
PERS, score between 3 and 4	**2.23 (1.14;4.36)**	**.0195**	**2.98 (1.31;6.79)**	**.0093**	2.10 (0.93;4.74)	.0758
PERS, score of 4 or higher	**5.18 (2.44;11.00)**	**<.0001**	**3.83 (1.53;9.61)**	**.0042**	**5.87 (2.54;13.54)**	**<.0001**

All analyses were adjusted for sex and age, significant results (p < 0.05) are reported in bold. * No longer significant after Benjamini–Hochberg correction.

Next, we computed a simple summed score of risk factors (ERS), which was associated with PE/PS at age 18. The simple summed score showed smaller ORs compared to the weighted PERS. The PERS was associated with PE and PS at follow-up (Table 3). Specifically, a PERS in the range 3-4 and a PERS of ≥ 4 were associated with higher odds of PE and PS, 2.2. and 5.2, where a higher PERS corresponded to higher odds.

### Sensitivity Analyses

Based on previous studies, we calculated 2 alternative aggregated risk scores.^12,13^ The PERS was highly correlated to both the P-PERS (*r* = .74, *P* < .001) and the M-PERS (*r* = .82, *P* < .001), which were moderately correlated with each other (*r* = .59, *P* < .001). As shown in Supplementary Table S2, the P-PERS was not significantly associated with PE/PS in our sample. The M-PERS showed lower ORs for PE/PS, PE, and PS compared to the 14-item PERS models. The M-PERS showed lower Nagelkerke pseudo-*R*^2^ of .026 to .028 compared to the PERS models that showed Nagelkerke pseudo-*R*^2^ of .031 to .043 which indicated a better fit but does not reflect the proportion of variance accounted for.^43^ When comparing the 3 aggregated risk scores, the current PERS demonstrated the best model fit as indicated by the lowest 2 log-likelihood and highest Nagelkerke pseudo-*R*^2^ values. Next, we examined whether the associations would remain if we excluded parental SMI from the PERS (Supplementary Table S3). For the continuous PERS, the ORs were almost identical to the ORs from the PERS that included parental SMI. For the dose–response analyses (ranges of PERS), 2 ORs for lower-scoring PERS scores were no longer significant. However, all other associations remained and showed similar ORs, for example, for PERS ≥ 4 ranging from 3.40 to 6.14, indicating that these associations were not primarily driven by genetic liability. Lastly, the linear regression analyses for the total score of PE showed the same associations, where a higher PERS was associated with a higher score on PE (Supplementary Table S4).

## Discussion

The present study combined 14 environmental risk factors for psychosis, measured at age 15, to examine their association with PE and PS at age 18. Although not all individual risk factors were associated with PE or PS in our sample, we found that the PERS was associated with higher odds of PE/PS in late adolescence. Specifically, a PERS between 3 and 4 was associated with a 2-fold increase of PE/PS, a score of 4 or higher was associated with a 5-fold increase of PE/PS later on. The updated and extended PERS exhibited a better model fit in our adolescent sample compared to 2 previously defined environmental risk scores based on fewer risk factors and different weightings.

A combined score of environmental risk factors has previously been shown to be associated with psychosis.^12,13,15–19^ Our results add to these findings that the PERS is also associated with subclinical symptoms. We examined both self-reported PE and clinician-rated PS, finding comparable results with stronger associations between the PERS and PS. The type of instrument used significantly influences the reported prevalence of PE, with structured interviews and questionnaires tending to overestimate prevalence compared to semistructured interviews.^44^ Notably, clinician-rated PE have shown to carry the highest risk for later psychiatric disorders.^45^ However, several studies have also found that false-positive PE—self-reported PE that are not confirmed by clinician ratings—, are still associated with an increased risk of later psychiatric disorders.^45,46^

Recently, a study found similar results for the PERS and PE in another western cohort that included adults from the general population,^22^ but a Brazilian study did not find an association with PE in high-risk adolescents.^23^ This discrepancy could be due to the specific sociocultural characteristics of the Brazilian cohort, underscoring the importance of a culturally sensitive approach when creating the PERS.^13,23^ This highlights the broader issue of a lack of population diversity in socio-environmental research, where most studies focus on WEIRD (Western, Educated, Industrialized, Rich, and Democratic) societies, leaving a significant portion of the global population unrepresented and creating substantial knowledge gaps.^47^ Alternatively, some risk factors might be predictive of psychosis, but not of subclinical psychotic symptoms, as we also did not find an association with that specific combination of risk factors in our study.

In the present study, all risk factors were assessed before the age of 15. Exposure to these risk factors in childhood and young adolescence was associated with subsequent psychotic symptoms in late adolescence. Furthermore, it was evident that the accumulation of risk factors was associated with later PE/PS, specifically a higher PERS was associated with higher odds. This underscores the dose–response effect of risk factors, demonstrating that the PERS effectively differentiates adolescents at high risk.^13,48^ Therefore, determining the PERS could aid general practitioners, early detection teams, and clinicians, in selecting adolescents for tailored prevention.^49,50^

The PERS as composed for the current study included 14 diverse risk factors, including perinatal factors, sociodemographic factors, various adverse life events, and parental factors. These risk factors have previously been shown to be associated with the risk for psychotic disorders.^30–37^ However, their specific contribution to the PERS score remains unknown. For example, parental severe mental illness not only has an environmental effect, for example, how the parent raises the child, but also a strong genetic component given the high heritability of severe mental illness.^51^ In our sensitivity analyses, we excluded parental severe mental illness from the PERS and found similar associations with similar effect sizes. This indicated that the effects were not primarily driven by genetic liability. Although it remains challenging to disentangle the biological and the environmental risk mechanisms, we included parental severe mental illness as this is a well-known risk factor, which is easier to assess than genetic risk scores and reflects an important part of the child’s environment.

In recent years, more studies have started to combine environmental risk factors using different approaches, ranging from simple sum scores,^20,24,52^ similar meta-analytical approaches,^12,13,16^ to modeling techniques.^17,53^ More advanced risk scores could perform slightly better and should focus on high sensitivity and specificity in prediction models for research to determine absolute risks.^54^ The currently adopted meta-analytical approach can be implemented more easily, allowing a clinician to rate the PERS to estimate the risk, an example is provided in the Supplementary Materials. Eventually, the PERS could function as a risk calculator, which has already been done for suicide,^55^ for the conversion from risk to psychosis,^56^ and for the risk of psychosis in non-psychotic patients.^16,57^

Interestingly, only if an adolescent had a PERS above 3 the risk exceeded those of the individual risk factors. The PERS could be a valuable tool in selecting adolescents at high risk of psychotic symptoms. However, more research is needed to compare this aggregated score to specific risk factors with a strong individual effect. The aggregated score does not allow for the identification of risk factors that disproportionally contribute to the overall score. To better determine the predictive utility, future studies should clarify whether it is more beneficial to focus on the highest individual risk factors or to leverage a combined score that captures the additive effects of multiple factors with lower individual impact.

We used a detailed risk score combining 14 risk factors, weighted with the most recent meta-analyses. Most studies used fewer risk factors, with the exception of O’Hare et al.,^24^ who combined 19 risk factors in children with schizotypy, including 8 perinatal risk factors. Compared to the previous 9-factor score from Padmanabhan et al.^12^ and 6-factor score from Vassos et al.,^13^ our updated PERS had a better model fit. This improved fit suggests that including a broader range of environmental risk factors and using updated weightings enhances the predictive accuracy and reliability of the PERS in identifying adolescents at risk for psychotic symptoms. It should be noted that previous studies could have chosen a PERS with fewer risk factors for easier administration. Specifically, the inclusion of parental severe mental illness warrants consideration as this factor encompasses both biological and environmental risk mechanisms. While the question of whether more risk factors are always better could be debated, the factors included collectively impact child development, regardless of the mechanisms through which they exert their influence. Future studies should aim to identify the optimal combination of risk factors. In addition, to gain an integrated understanding of the interplay of risk factors, future research should also include the PRS, given the gene-environmental interactions previously described.^48,58^

The current study possesses several notable strengths. By using detailed phenotype data measured in a high-risk cohort of adolescents, we were able to investigate 14 risk factors and their associations with both self-reported psychotic experiences and clinician-rated psychotic symptoms. The prevalence rates of both PE and PS were high in this population-based at-risk cohort which provided sufficient statistical power to study these associations. Furthermore, the use of longitudinal data made it possible to investigate whether the PERS, measured with lifetime risk factors assessed at age 15, was predictive of PE and PS at age 18. This longitudinal approach enhances the robustness of our findings, offering insights into the relationship between early risk factors and subsequent psychotic outcomes.

However, the study also had some limitations. The PERS was calculated using ORs specific for psychotic disorders. For some risk factors, it would also be possible to consider ORs specific for psychotic experiences,^59–61^ or perhaps even broader for psychopathology.^62^ The current results focused on the association between psychosis risk factors and subclinical symptoms. Selecting the ORs must be determined based on the specific purpose for which the PERS is applied. Furthermore, the combination of risk factors and their binarized cut-off depended on both the available meta-analyses as well as the available data in the cohort. For some factors, including physical and sexual abuse, we solely relied on the parent-reported data available, which could potentially lead to underestimation compared to self-report data. We were unable to include other relevant risk factors such as hearing impairment,^63^ immigration,^7^ or neglect.^35^ The use of binarized risk factors also does not take into account specific categories, for example, for ethnicity, or specific dose–response effects of cannabis use or urbanicity. While the 14-item score is advantageous in capturing a comprehensive range of risk factors, its complexity may hinder implementation as it requires detailed and time-intensive assessment in both clinical and also research contexts. Lastly, although the PERS is a sophisticated method of combining several risk factors into one score, it does not consider the small correlations between risk factors in our sample nor does it take into account possible interaction effects. Our results are limited by assuming an additive effect of risk factors, which is likely to be a simplification of the complex interplay between risk factors. This limitation underscores the need for the development of more nuanced models that can better capture the intricacies of risk factor interactions.

In conclusion, this study demonstrated that a detailed, weighted environmental risk score measured in early adolescence, the PERS, is associated with psychotic experiences and symptoms 3 years later in late adolescence. The findings support the utility of combining multiple risk factors to improve the prediction of psychotic experiences and symptoms. The PERS could be a valuable tool for identifying adolescents at high risk for psychosis and informing targeted prevention strategies and early interventions. Further research is needed to refine the PERS, explore its application in diverse populations, and integrate genetic risk factors to enhance its predictive power.

## Supplementary Material

Supplementary material is available at https://academic.oup.com/schizophreniabulletin.

sbaf046_suppl_Supplementary_Tables_S1-S4

## References

[1] Kim JY, Son MJ, Son CY, et al Environmental risk factors and biomarkers for autism spectrum disorder: an umbrella review of the evidence. Lancet Psychiatry. 2019;6:590–600. 31230684 10.1016/S2215-0366(19)30181-6

[2] Kim JH, Kim JY, Lee J, et al Environmental risk factors, protective factors, and peripheral biomarkers for ADHD: an umbrella review. Lancet Psychiatry. 2020;7:955–970. 33069318 10.1016/S2215-0366(20)30312-6

[3] Hazzard VM, Mason TB, Smith KE, et al Identifying transdiagnostically relevant risk and protective factors for internalizing psychopathology: an umbrella review of longitudinal meta-analyses. J Psychiatr Res. 2023;158:231–244. 36603318 10.1016/j.jpsychires.2022.12.025 PMC9898156

[4] Bortolato B, Köhler CA, Evangelou E, et al Systematic assessment of environmental risk factors for bipolar disorder: an umbrella review of systematic reviews and meta-analyses. Bipolar Disord. 2017;19:84–96. 28470927 10.1111/bdi.12490

[5] Fullana MA, Tortella-Feliu M, Fernández de la Cruz L, et al Risk and protective factors for anxiety and obsessive-compulsive disorders: an umbrella review of systematic reviews and meta-analyses. Psychol Med. 2020;50:1300–1315. 31172897 10.1017/S0033291719001247

[6] Solmi M, Dragioti E, Arango C, et al Risk and protective factors for mental disorders with onset in childhood/adolescence: an umbrella review of published meta-analyses of observational longitudinal studies. Neurosci Biobehav Rev. 2021;120:565–573. 32931804 10.1016/j.neubiorev.2020.09.002

[7] Radua J, Ramella-Cravaro V, Ioannidis JPA, et al What causes psychosis? An umbrella review of risk and protective factors. World Psychiatry. 2018;17:49–66. 29352556 10.1002/wps.20490 PMC5775150

[8] Belbasis L, Köhler CA, Stefanis N, et al Risk factors and peripheral biomarkers for schizophrenia spectrum disorders: an umbrella review of meta-analyses. Acta Psychiatr Scand. 2018;137:88–97. 29288491 10.1111/acps.12847

[9] Krantz MF, Ellersgaard D, Andersen KK, et al Accumulation of disadvantages across multiple domains amongst subgroups of children of parents with schizophrenia or bipolar disorder: clustering data from the danish high risk and resilience study VIA 7. Schizophr. Bull. Open. 2022;3:sgac010. 39144761 10.1093/schizbullopen/sgac010 PMC11206045

[10] Rodriguez V, Alameda L, Trotta G, et al Environmental risk factors in bipolar disorder and psychotic depression: a systematic review and meta-analysis of prospective studies. Schizophr Bull. 2021;47:959–974. 33479726 10.1093/schbul/sbaa197 PMC8266635

[11] McGorry PD, Hartmann JA, Spooner R, Nelson B. Beyond the “at risk mental state” concept: transitioning to transdiagnostic psychiatry. World Psychiatry. 2018;17:133–142. 29856558 10.1002/wps.20514 PMC5980504

[12] Padmanabhan JL, Shah JL, Tandon N, Keshavan MS. The “polyenviromic risk score”: aggregating environmental risk factors predicts conversion to psychosis in familial high-risk subjects. Schizophr Res. 2017;181:17–22. 28029515 10.1016/j.schres.2016.10.014 PMC5365360

[13] Vassos E, Sham P, Kempton M, et al The Maudsley environmental risk score for psychosis. Psychol Med. 2020;50:2213–2220. 31535606 10.1017/S0033291719002319 PMC7557157

[14] Lewis CM, Vassos E. Polygenic risk scores: from research tools to clinical instruments. Genome Med. 2020;12:44. 32423490 10.1186/s13073-020-00742-5 PMC7236300

[15] Oliver D, Radua J, Reichenberg A, Uher R, Fusar-Poli P. Psychosis Polyrisk Score (PPS) for the detection of individuals at-risk and the prediction of their outcomes. review. Front Psychiatry. 2019;10:174. 31057431 10.3389/fpsyt.2019.00174 PMC6478670

[16] Oliver D, Spada G, Englund A, et al Real-world digital implementation of the Psychosis Polyrisk Score (PPS): a pilot feasibility study. Schizophr Res. 2020;226:176–183. 32340785 10.1016/j.schres.2020.04.015 PMC7774585

[17] Pries L-K, Lage-Castellanos A, Delespaul P, et al; Genetic Risk and Outcome of Psychosis (GROUP) investigators. Estimating exposome score for schizophrenia using predictive modeling approach in two independent samples: the results from the EUGEI Study. Schizophr Bull. 2019;45:960–965. 31508804 10.1093/schbul/sbz054 PMC6737483

[18] Pries LK, Erzin G, van Os J, et al Predictive performance of exposome score for schizophrenia in the general population. Schizophr Bull. 2021;47:277–283. 33215211 10.1093/schbul/sbaa170 PMC7965069

[19] Jeon EJ, Kang SH, Piao YH, et al Development of the Korea-Polyenvironmental Risk Score for psychosis. Psychiatry Investig. 2022;19:197–206. 10.30773/pi.2021.0328 PMC8958209 35196829

[20] Stepniak B, Papiol S, Hammer C, et al Accumulated environmental risk determining age at schizophrenia onset: a deep phenotyping-based study. Lancet Psychiatry. 2014;1:444–453. 26361199 10.1016/S2215-0366(14)70379-7

[21] Erzin G, Pries LK, Dimitrakopoulos S, et al Association between exposome score for schizophrenia and functioning in first-episode psychosis: results from the Athens first-episode psychosis research study. Psychol Med. 2023;53:2609–2618. 34789350 10.1017/S0033291721004542 PMC10123830

[22] Rejek M, Misiak B. Modelling the effects of the exposome score within the extended psychosis phenotype. Article. J Psychiatr Res. 2024;169:22–30. 37995498 10.1016/j.jpsychires.2023.11.022

[23] Navarro GOSV, Fonseca L, Talarico F, et al Polyenvironmental and polygenic risk scores and the emergence of psychotic experiences in adolescents. J Psychiatr Res. 2021;142:384–388. 34450553 10.1016/j.jpsychires.2021.07.057

[24] O’Hare K, Watkeys O, Whitten T, et al Cumulative environmental risk in early life: associations with schizotypy in childhood. Schizophr Bull. 2022;49:244–254. 10.1093/schbul/sbac160 PMC10016419 36302227

[25] Goodman R. Psychometric properties of the strengths and difficulties questionnaire. J Am Acad Child Adolesc Psychiatry. 2001;40:1337–1345. 11699809 10.1097/00004583-200111000-00015

[26] Grootendorst-van Mil NH, Bouter DC, Hoogendijk WJG, et al The iBerry study: a longitudinal cohort study of adolescents at high risk of psychopathology. Eur J Epidemiology. 2021;36:453–464. 10.1007/s10654-021-00740-w PMC8076148 33796978

[27] Bouter DC, Ravensbergen SJ, de Neve-Enthoven NGM, et al Five-year follow-up of the iBerry Study: screening in early adolescence to identify those at risk of psychopathology in emerging adulthood. Eur Child Adolesc Psychiatry. 2024;33:4285–4294. 38772966 10.1007/s00787-024-02462-2 PMC11618212

[28] Ising HK, Veling W, Loewy RL, et al The validity of the 16-item version of the Prodromal Questionnaire (PQ-16) to screen for ultra high risk of developing psychosis in the general help-seeking population. Schizophr Bull. 2012;38:1288–1296. 22516147 10.1093/schbul/sbs068 PMC3713086

[29] Sheehan DV, Sheehan KH, Shytle RD, et al Reliability and validity of the Mini International Neuropsychiatric Interview for Children and Adolescents (MINI-KID). J Clin Psychiatry. 2010;71:313–326. 20331933 10.4088/JCP.09m05305whi

[30] Davies C, Segre G, Estradé A, et al Prenatal and perinatal risk and protective factors for psychosis: a systematic review and meta-analysis. Lancet Psychiatry. 2020;7:399–410. 32220288 10.1016/S2215-0366(20)30057-2

[31] Henssler J, Brandt L, Müller M, et al Migration and schizophrenia: meta-analysis and explanatory framework. Eur Arch Psychiatry Clin Neurosci. 2020;270:325–335. 31161262 10.1007/s00406-019-01028-7

[32] Vassos E, Pedersen CB, Murray RM, Collier DA, Lewis CM. Meta-analysis of the association of urbanicity with schizophrenia. Schizophr Bull. 2012;38:1118–1123. 23015685 10.1093/schbul/sbs096 PMC3494055

[33] Marconi A, Di Forti M, Lewis CM, Murray RM, Vassos E. Meta-analysis of the association between the level of cannabis use and risk of psychosis. Schizophr Bull. 2016;42:1262–1269. 26884547 10.1093/schbul/sbw003 PMC4988731

[34] Pastore A, de Girolamo G, Tafuri S, Tomasicchio A, Margari F. Traumatic experiences in childhood and adolescence: a meta-analysis of prospective studies assessing risk for psychosis. Eur Child Adolesc Psychiatry. 2022;31:215–228. 32577908 10.1007/s00787-020-01574-9

[35] Varese F, Smeets F, Drukker M, et al Childhood adversities increase the risk of psychosis: a meta-analysis of patient-control, prospective- and cross-sectional cohort studies. Schizophr Bull. 2012;38:661–671. 22461484 10.1093/schbul/sbs050 PMC3406538

[36] Ayerbe L, Pérez-Piñar M, Foguet-Boreu Q, Ayis S. Psychosis in children of separated parents: a systematic review and meta-analysis. Eur Psychiatry. 2020;63:e3. 32093793 10.1192/j.eurpsy.2019.15 PMC7315852

[37] Rasic D, Hajek T, Alda M, Uher R. Risk of mental illness in offspring of parents with schizophrenia, bipolar disorder, and major depressive disorder: a meta-analysis of family high-risk studies. Schizophr Bull. 2014;40:28–38. 23960245 10.1093/schbul/sbt114 PMC3885302

[38] Straus MA, Mattingly MJ. A Short Form and Severity Level Types for the Parent-Child Conflict Tactics Scales. Family Research Laboratory, University of New Hampshire; 2007.

[39] Ormel J, Oldehinkel AJ, Sijtsema J, et al The TRacking Adolescents’ Individual Lives Survey (TRAILS): design, current status, and selected findings. J Am Acad Child Adolesc Psychiatry. 2012;51:1020–1036. 23021478 10.1016/j.jaac.2012.08.004

[40] Sheehan DV, Lecrubier Y, Sheehan KH, et al The Mini-International Neuropsychiatric Interview (M.I.N.I.): the development and validation of a structured diagnostic psychiatric interview for DSM-IV and ICD-10. J Clin Psychiatry. 1998;59:22–33;quiz 34. 9881538

[41] White IR, Royston P, Wood AM. Multiple imputation using chained equations: issues and guidance for practice. Stat Med. 2011;30:377–399. 21225900 10.1002/sim.4067

[42] IBM Corp. IBM SPSS Statistics for Windows, version 28.0. Armonk, NY: IBM Corp.; 2021.

[43] Cohen J, Cohen P, West SG, Aiken LS. Applied Multiple Regression/Correlation Analysis for the Behavioral Sciences. 3rd ed. Lawrence Erlbaum Associates Publishers; 2003:703–70xxviii.

[44] Monshouwer K, ten Have M, Tuithof M, et al Prevalence, incidence, and persistence of psychotic experiences in the general population: results of a 9-year follow-up study. Psychol Med. 2022;53:3750–3761. 36117284 10.1017/S0033291722002690

[45] Rimvall MK, Gundersen S, Clemmensen L, et al Evidence that self-reported psychotic experiences in children are clinically relevant. Schizophr Res. 2019;204:415–416. 30121187 10.1016/j.schres.2018.08.003

[46] van Nierop M, van Os J, Gunther N, et al Phenotypically continuous with clinical psychosis, discontinuous in need for care: evidence for an extended psychosis phenotype. Schizophr Bull. 2011;38:231–238. 21908795 10.1093/schbul/sbr129 PMC3283149

[47] Burkhard C, Cicek S, Barzilay R, Radhakrishnan R, Guloksuz S. Need for ethnic and population diversity in psychosis research. Schizophr Bull. 2021;47:889–895. 33948664 10.1093/schbul/sbab048 PMC8266627

[48] Mas S, Boloc D, Rodríguez N, et al Examining gene–environment interactions using aggregate scores in a first-episode psychosis cohort. Schizophr Bull. 2020;46:1019–1025. 32083289 10.1093/schbul/sbaa012 PMC7342095

[49] Fusar-Poli P, Correll CU, Arango C, Berk M, Patel V, Ioannidis JPA. Preventive psychiatry: a blueprint for improving the mental health of young people. World Psychiatry. 2021;20:200–221. 34002494 10.1002/wps.20869 PMC8129854

[50] Salazar de Pablo G, De Micheli A, Solmi M, et al Universal and selective interventions to prevent poor mental health outcomes in young people: systematic review and meta-analysis. Harv Rev Psychiatry. 2021;29:196–215. 33979106 10.1097/HRP.0000000000000294

[51] Polderman TJC, Benyamin B, de Leeuw CA, et al Meta-analysis of the heritability of human traits based on fifty years of twin studies. Nat Genet. 2015;47:702–709. 25985137 10.1038/ng.3285

[52] Newbury JB, Arseneault L, Caspi A, et al Association between genetic and socioenvironmental risk for schizophrenia during upbringing in a UK longitudinal cohort. Psychol Med. 2020;52:1527–1537. 32972469 10.1017/S0033291720003347 PMC9226384

[53] Pries LK, Moore TM, Visoki E, Sotelo I, Barzilay R, Guloksuz S. Estimating the association between exposome and psychosis as well as general psychopathology: results from the ABCD study. Biol Psychiatry Glob Open Sci. 2022;2:283–291. 36325038 10.1016/j.bpsgos.2022.05.005 PMC9616253

[54] Pries LK, Erzin G, Rutten BPF, van Os J, Guloksuz S. Estimating aggregate environmental risk score in psychiatry: the exposome score for schizophrenia. Front Psychiatry. 2021;12:671334. 34122186 10.3389/fpsyt.2021.671334 PMC8193078

[55] Sariaslan A, Fanshawe T, Pitkänen J, Cipriani A, Martikainen P, Fazel S. Predicting suicide risk in 137,112 people with severe mental illness in Finland: external validation of the Oxford Mental Illness and Suicide tool (OxMIS). Transl Psychiatry. 2023;13:126. 37072392 10.1038/s41398-023-02422-5 PMC10113231

[56] Cannon TD, Yu C, Addington J, et al. An individualized risk calculator for research in prodromal psychosis. Am J Psychiatry. 2016;173:980–988. 27363508 10.1176/appi.ajp.2016.15070890 PMC5048498

[57] Fusar-Poli P, Rutigliano G, Stahl D, et al Development and validation of a clinically based risk calculator for the transdiagnostic prediction of psychosis. JAMA Psychiatry. 2017;74:493–500. 28355424 10.1001/jamapsychiatry.2017.0284 PMC5470394

[58] Guloksuz S, Rutten BPF, Pries L-K, et al; European Network of National Schizophrenia Networks Studying Gene-Environment Interactions Work Package 6 (EU-GEI WP6) Group. The complexities of evaluating the exposome in psychiatry: a data-driven illustration of challenges and some propositions for amendments. Schizophr Bull. 2018;44:1175–1179. 30169883 10.1093/schbul/sby118 PMC6192470

[59] Matheson SL, Laurie M, Laurens KR. Substance use and psychotic-like experiences in young people: a systematic review and meta-analysis. Psychol Med. 2023;53:305–319. 36377500 10.1017/S0033291722003440 PMC9899577

[60] Groening JM, Denton E, Parvaiz R, et al A systematic evidence map of the association between cannabis use and psychosis-related outcomes across the psychosis continuum: an umbrella review of systematic reviews and meta-analyses. Psychiatry Res. 2024;331:115626. 38096722 10.1016/j.psychres.2023.115626

[61] Leaune E, Dealberto M-J, Luck D, et al Ethnic minority position and migrant status as risk factors for psychotic symptoms in the general population: a meta-analysis. Psychol Med. 2019;49:545–558. 30178719 10.1017/S0033291718002271

[62] Arango C, Dragioti E, Solmi M, et al Risk and protective factors for mental disorders beyond genetics: an evidence-based atlas. World Psychiatry. 2021;20:417–436. 34505386 10.1002/wps.20894 PMC8429329

[63] Linszen MM, Brouwer RM, Heringa SM, Sommer IE. Increased risk of psychosis in patients with hearing impairment: review and meta-analyses. Neurosci Biobehav Rev. 2016;62:1–20. 26743858 10.1016/j.neubiorev.2015.12.012

