# Supplementary material for: Combining the Risk: The Poly-Environmental Risk Score and Psychotic Symptoms in Adolescents
Source: Schizophr Bull. 2025 Apr 14;51(5):1464–72. doi: 10.1093/schbul/sbaf046 (PMC12414554; doi:10.1093/schbul/sbaf046)
Supplement: sbaf046_suppl_Supplementary_Tables_S1-S4 [file sbaf046_suppl_supplementary_tables_s1-s4.docx]

# Supplementary tables

| **Supplementary table S1.** Pearson correlation coefficients for the 14 dichotomized risk factors used in the poly-environmental risk score | | | | | | | | | | | | | | | | | | | | | | |
| --- | --- | --- | --- | --- | --- | --- | --- | --- | --- | --- | --- | --- | --- | --- | --- | --- | --- | --- | --- | --- | --- | --- |
|  | | | 1. | 2. | | 3. | | 4. | 5. | | 6. | | 7. | 8. | | 9. | | | 10. | 11. | 12. | 13. |
| 1. Winter birth | | | * |  | |  | |  |  | |  | |  |  | |  | | |  |  |  |  |
| 2. Gestational age | | | **.106, *p*=.0109** | * | |  | |  |  | |  | |  |  | |  | | |  |  |  |  |
| 3. Birth weight | | | **.102, *p*=.0180** | **.570, *p*=<.0001** | | * | |  |  | |  | |  |  | |  | | |  |  |  |  |
| 4. Ethnic minority status | | | .015, *p*=.6673 | .074, *p*=.0775 | | .042, *p*=.3288 | | * |  | |  | |  |  | |  | | |  |  |  |  |
| 5. Urban living area | | | .026, *p*=.4651 | .027, *p*=.5187 | | -.038, *p*=.3812 | | **.168, *p*=<.0001** | * | |  | |  |  | |  | | |  |  |  |  |
| 6. Used cannabis | | | .012, *p*=.7331 | -.016, *p*=.7011 | | -.031, *p*=.4728 | | -.018, *p*=.6192 | .004, *p*=.9197 | | * | |  |  | |  | | |  |  |  |  |
| 7. Has been bullied | | | .007, *p*=.8460 | -.006, *p*=.8906 | | -.011, *p*=.8068 | | -.009, *p*=.7986 | .013, *p*=.7281 | | .046, *p*=.2195 | | * |  | |  | | |  |  |  |  |
| 8. Emotional abuse | | | .023, *p*=.5257 | .047, *p*=.2610 | | -.037, *p*=.3902 | | **.146, *p*=<.0001** | -.006, *p*=.8763 | | .049, *p*=.1797 | | **.129, *p*=.0005** | * | |  | | |  |  |  |  |
| 9. Physical abuse | | | .041, *p*=.2715 | .029, *p*=.4905 | | .049, *p*=.2632 | | .050, *p*=.1837 | .027, *p*=.4702 | | **.147, *p*=<.0001** | | **.141, *p*=.0002** | **.114, *p*=.0025** | | * | | |  |  |  |  |
| 10. Sexual abuse | | | -.046, *p*=.2170 | .018, *p*=.6665 | | **.098, *p*=.025** | | .007, *p*=.8418 | -.036, *p*=.3309 | | .069, *p*=.0673 | | **.095, *p*=.0137** | .052, *p*=.1697 | | **.130, *p*=.0006** | | | * |  |  |  |
| 11. Paternal age | | | .021, *p*=.6341 | **.105, *p*=.0287** | | .093, *p*=.0602 | | .034, *p*=.4377 | -.082, *p*=.0619 | | .081, *p*=.0671 | | .015, *p*=.7317 | -.042, *p*=.3446 | | -.048, *p*=.2847 | | | -.039, *p*=.3874 | * |  |  |
| 12. Parental divorce | | | -.043, *p*=.2443 | .074, *p*=.0773 | | -.020, *p*=.6478 | | **.102, *p*=.0057** | **.078, *p*=.0344** | | **.103, *p*=.0052** | | .052, *p*=.1702 | .031, *p*=.4139 | | **.131, *p*=.0005** | | | .050, *p*=.1809 | -.082, *p*=.0652 | * |  |
| 13. Parental severe mental   illness | | | -.027, *p*=.4766 | -.036, *p*=.3911 | | -.046, *p*=.2894 | | -.060, *p*=.1061 | .028, *p*=.4602 | | -.023, *p*=.5441 | | .060, *p*=.1153 | .051, *p*=.1797 | | .052, *p*=.1696 | | | .066, *p*=.0809 | -.065, *p*=.1453 | **.084, *p*=.0243** | * |
| 14. Parental death | | | -.008, *p*=.8391 | -.026, *p*=.5383 | | -.025, *p*=.5683 | | .041, *p*=.2628 | -.026, *p*=.4783 | | -.023, *p*=.5387 | | -.033, *p*=.3882 | -.011, *p*=.7614 | | -.014, *p*=.7138 | | | .044, *p*=.2359 | n.a. | .019, *p*=.6026 | **.039, *p*=.2961** |
| **Supplementary table S2.** ORs from logistic regression analyses for the two different PERSs from previous studies | | | | | | | | | | | | | | | |  |  |  |  |  |  |  |
|  | **Total PE/PS group (n=801)** | | | | | **Psychotic experiences (n=791)** | | | | | **Psychotic symptoms (n=679)** | | | | |  |  |  |  |  |  |  |
|  | **OR (95% CI)** | | | ***p*-value** | | **OR (95% CI)** | | | ***p*-value** | | **OR (95% CI)** | | | ***p*-value** | | |  |  |  |  |  |  |
| P-PERS^1^ |  | | |  | |  | | |  | |  | | |  | | |  |  |  |  |  |  |
| Constant | 5.29 (0.12;226.46) | | | .3848 | | 4.39 (0.05;382.78) | | | .5169 | | 0.01 (0.00;1.78) | | | .0838 | | |  |  |  |  |  |  |
| Age | 0.84 (0.68;1.03) | | | .0970 | | 0.82 (0.64;1.05) | | | .1170 | | 1.13 (0.86;1.48) | | | .3826 | | |  |  |  |  |  |  |
| Sex | 1.00 (0.71;1.41) | | | .9834 | | 1.30 (0.87;1.95) | | | .1995 | | 1.18 (0.78;1.77) | | | .4419 | | |  |  |  |  |  |  |
| P-PERS | 1.26 (0.98;1.61) | | | .0727 | | 1.22 (0.91;1.64) | | | .1806 | | 1.32 (0.99;1.77) | | | .0625 | | |  |  |  |  |  |  |
|  |  | | |  | |  | | |  | |  | | |  | | |  |  |  |  |  |  |
| M-PERS^2^ |  | | |  | |  | | |  | |  | | |  | | |  |  |  |  |  |  |
| Constant | 11.27 (0.25;517.19) | | | .2148 | | 9.68 (0.10;920.93) | | | .3286 | | 0.04 (0.00;5.22) | | | .1914 | | |  |  |  |  |  |  |
| Age | 0.81 (0.65;1.00) | | | .0504 | | 0.79 (0.61;1.02) | | | .0652 | | 1.08 (0.82;1.42) | | | .5936 | | |  |  |  |  |  |  |
| Sex | 1.00 (0.71;1.41) | | | .9994 | | 1.30 (0.87;1.95) | | | .1981 | | 1.17 (0.78;1.77) | | | .4493 | | |  |  |  |  |  |  |
| M-PERS | **1.10 (1.04;1.17)** | | | **.0014** | | **1.10 (1.03;1.18)** | | | **.0072** | | **1.12 (1.04;1.120)** | | | **.0026** | | |  |  |  |  |  |  |
|  |  | | |  | |  | | |  | |  | | |  | | |  |  |  |  |  |  |
| PERS^3^ | | | | | | | | |  | |  | | |  | | |  |  |  |  |  |  |
| Constant | 9.39 (0.20;435.89) | | | .2528 | | 7.14 (0.08;667.04) | | | .3957 | | 0.03 (0.00;4.32) | | | .1671 | | |  |  |  |  |  |  |
| Age | 0.79 (0.64;0.98) | | | .0335 | | 0.78 (0.61;1.01) | | | .0572 | | 1.05 (0.80;1.39) | | | .7309 | | |  |  |  |  |  |  |
| Sex | 1.01 (0.71;1.42) | | | .9767 | | 1.31 (0.87;1.96) | | | .1946 | | 1.18 (0.78;1.79) | | | .4288 | | |  |  |  |  |  |  |
| PERS | **1.38 (1.20;1.60)** | | | **<.0001** | | **1.32 (1.12;1.55)** | | | **.0011** | | **1.45 (1.22;1.72)** | | | **<.0001** | | |  |  |  |  |  |  |

^1^ P-PERS is based on the study by Padmanabhan (2017,^12^), calculated by summing the log odds for winter or spring birth (0.068), birth weight ≤ 2.5 kg (0.69), urban living (0.54), cannabis use (0.56), physical abuse (1.08), sexual abuse (0.87), paternal age over 35 years (0.25), and parental death (0.53). Neglect was not included as a risk factor, as data on the subject was not available.

^2^ M-PERS (The Maudsley ERS, 2020,^13^) was calculated by summing six risk factors. We summed ethnic minority status (+2.5) or non-minority status (as native, -0.5), urbanicity low (-1.5) or high (+1), paternal age 40-50 years (+0.5), birth weight ≤ 2.5 kg (+2), cannabis exposure (no exposure, -1; exposure, +3), and any childhood adversity (+2.5).

^3^ Included for comparison of the models, results are also reported in Table 3.

|  | **Total PE/PS group**  **(n=801)** | | **Psychotic experiences (n=791)** | | **Psychotic symptoms (n=679)** | |
| --- | --- | --- | --- | --- | --- | --- |
|  | OR (95% CI) | *p* | OR (95% CI) | *p* | OR (95% CI) | *p* |
| **Risk scores without parental severe mental illness** | | |  |  |  |  |
| ERS (simple sum) | **1.20 (1.08;1.34)** | **.0008** | **1.16 (1.03;1.32)** | **.0189** | **1.24 (1.09;1.42)** | **.0011** |
|  |  |  |  |  |  |  |
| PERS (continuous) | **1.37 (1.18;1.59)** | **<.0001** | **1.30 (1.10;1.53)** | **.0023** | **1.45 (1.22;1.72)** | **<.0001** |
|  |  |  |  |  |  |  |
| PERS, score between 1-2 | 1.37 (0.80;2.32) | .2495 | 1.76 (0.94;3.30) | .0756 | 1.17 (0.61;2.27) | .6372 |
| PERS, score between 2-3 | 1.34 (0.76;2.34) | .3101 | 1.63 (0.82;3.28) | .1664 | 1.35 (0.69;2.66) | .3836 |
| PERS, score between 3-4 | 1.93 (0.99;3.76) | .0538 | **2.38 (1.06;5.38)** | **.0366** | 2.01 (0.93;4.36) | .0763 |
| PERS, score of 4 or higher | **5.13 (2.43;10.84)** | **<.0001** | **3.40 (1.40;8.26)** | **.0069** | **6.14 (2.71;13.89)** | **<.0001** |

**Supplementary table S3.** Sensitivity analyses to examine the PERS without parental severe mental illness, ORs from logistic regression analyses for a combined simple sum score and the PERS in association with psychotic experiences and psychotic symptoms in adolescents

**Supplementary table S4.** Coefficients from linear regression analyses for a combined simple sum score and the PERS in association with the total score of psychotic experiences

|  | **Total PE** | |
| --- | --- | --- |
|  | **(n=791)** | |
|  | B (95% CI) | *p* |
| ERS (simple sum) | **0.07 (0.03;0.11)** | **.0003** |
|  |  |  |
| PERS (continuous) | **0.13 (0.07;0.18)** | **.0000** |
|  |  |  |
| PERS. score between 1-2 | 0.17 (-0.01;0.35) | .0536 |
| PERS. score between 2-3 | **0.26 (0.07;0.45)** | **.0083** |
| PERS. score between 3-4 | **0.40 (0.15;0.65)** | **.0018** |
| PERS. score of 4 or higher | **0.54 (0.23;0.85)** | **.0007** |

*Note. All analyses were adjusted for sex and age.*

*The PE sum score was transformed using a square root transformation to approximate normality.*

**Example of calculating the PERS**

| **Risk factor** | **OR** | **Log OR** |
| --- | --- | --- |
| Winter birth | 1.05 | 0.049 |
| Low gestational age | 1.35 | 0.300 |
| Low birth weight | 1.53 | 0.425 |
| Ethnic minority status | 1.82 | 0.599 |
| Urban living area | 2.39 | 0.871 |
| Used cannabis | 1.97 | 0.678 |
| Has been bullied | 2.28 | 0.824 |
| Emotional abuse | 3.40 | 1.224 |
| Physical abuse | 2.95 | 1.082 |
| Sexual abuse | 2.38 | 0.867 |
| High paternal age | 1.28 | 0.247 |
| Parental divorce | 1.53 | 0.425 |
| Parental severe mental illness | 3.94 | 1.371 |
| Parental death | 1.24 | 0.215 |

To calculate the PERS one sums the log OR for the present risk factors. absent risk factors can be skipped as log OR x 0 = 0.

For example. if someone reports four risk factors (low birth weight. living in an urban area. used cannabis and parental divorce) the PERS can be calculated by summing those log OR:

0.425 + 0.871 + 0.678 + 0.425 = 2.399

This score assumes that the other 10 factors were assessed but absent. If for three factors it was unknown whether this risk was present or absent it is possible to slightly adjust the score by multiplying the PERS with the total sum of the log OR for the 14-item PERS (9.177) divided by the sum of the log OR of the known risk factors.

For example. if the three abuse risk factors were unknown (sum of log OR: 1.224+1.082+0.867) the PERS score for 11 assessed risk factors assessed would be:

2.399 * (9.177 / 6.004) = 3.667

If for research purposes some measurements are absent completely from the data collection. the total sum of the OR could be adjusted accordingly.
